# The Domestic Environment and the Lung Mycobiome

**DOI:** 10.3390/microorganisms8111717

**Published:** 2020-11-02

**Authors:** Esther Rubio-Portillo, David Orts, Eleuterio Llorca, Cleofé Fernández, Josefa Antón, Consuelo Ferrer, Beatriz Gálvez, Violeta Esteban, Elena Revelles, Carlos Pérez-Martín, Enrique Gómez-Imbernón, Jorge Adsuar, Pedro Piqueras, Beatriz Amat, José Franco, María Francisca Colom

**Affiliations:** 1 Department of Physiology, Genetics and Microbiology, University of Alicante, Sant Vicent del Raspeig, 03690 Alicante, Spain; esther.portillo@ua.es (E.R.-P.); anton@ua.es (J.A.); 2 Pneumology Service, General University Hospital, Elda, 03600 Alicante, Spain; ortsorts@gmail.com (D.O.); ellorcam@coma.es (E.L.); 3 Respiratory Department, General University Hospital, Institute for Healthcare and Biomedical Research of Alicante (ISABIAL), 03010 Alicante, Spain; CleofeFernandez@hotmail.com; 4 Medical Mycology Laboratory, Department of Plant Production and Microbiology, Institute for Healthcare and Biomedical Research of Alicante (ISABIAL), Campus of Sant Joan d’Alacant, University Miguel Hernández, 03550 Alicante, Spain; c.ferrer@goumh.umh.es (C.F.); elenarv291@gmail.com (E.R.); carpermartin@gmail.com (C.P.-M.); enrgomez95@gmail.com (E.G.-I.); jorgeadsuar@gmail.com (J.A.); pedropiqueras14@gmail.com (P.P.); 5 Pneumology Department, Vinalopó University Hospital, Elche 03293 Alicante, Spain; bgalvez@vinaloposalud.com (B.G.); beatrizamat@hotmail.com (B.A.); 6 Respiratory Department, University Hospital of Valencia, 46010 Valencia, Spain; Violeta_ER@hotmail.com (V.E.); franco_jos@gva.es (J.F.)

**Keywords:** fungi, mycobiome, lower respiratory tract, house dust, mycobiota, bronco-alveolar lavage

## Abstract

This study analyzes the relationship between the mycobiome of the Lower Respiratory Tract (LRT) and the fungi in the domestic environment. Samples studied consisted of Broncho-Alveolar Lavage (BAL) from 45 patients who underwent bronchoscopy for different diagnostic purposes, and dust and air from the houses (ENV) of 20 of them (44.4%). Additionally, five bronchoscopes (BS) were also analyzed and negative controls were included for every procedure. All samples were processed for DNA extraction and cultures, which were performed in Sabouraud Dextrose and Potato Dextrose Agar. The fungal Internal Transcribed Spacer (ITS2) was sequenced by the Solexa/Illumina system and sequences were analyzed by QIIME 1.8.0 and compared with the UNITE Database for identification. The similarity between the two fungal communities (BAL and ENV) for a specific patient was assessed via the percentage of coincidence in the detection of specific operational taxonomic units (OTUs), and about 75% of co-occurrence was detected between the mycobiome of the LRT and the houses. Cultures confirmed the presence of the core mycobiome species. However, the low rate of isolation from BAL suggests that most of its mycobiome corresponds to non-culturable cells. This likely depends on the patient’s immune system activity and inflammatory status.

## 1. Introduction

High-throughput sequencing-based methods (HTS) are currently providing a wealth of information about the composition of the human microbiota, now considered an important “organ” playing a key role in maintaining the homeostasis of the body [1,2]. The gut microbiome is the most explored organ by far, but the study of other epithelial tissues such as those of the respiratory tract has also shown that their microbial communities are directly involved in in people’s health status of individuals [2,3,4,5,6]. Therefore, the microbiome of the respiratory tract is a topic of growing interest.

Few studies based on HTS have been performed to describe the lung mycobiome, that is, the study of fungi as members of the microbial community of the lungs. It is presumed that the species comprising that mycobiome reflect exposure to environmental and endogenous fungi [7,8,9], but this fact has not been explored deeply or specifically yet. In fact, until recently, the Lower Respiratory Tract (LRT) was believed to be sterile and this idea only changed after several studies demonstrated the existence of a microbial community in the lower airway of healthy individuals [10]. Nevertheless, in recent years, interesting contributions have shown a relationship between the airway mycobiome and the health status in patients with chronic inflammatory respiratory diseases. Thus, studies of patients suffering from cystic fibrosis (CF), asthma, and chronic obstructive pulmonary disease (COPD) demonstrate that fungal diversity is inversely proportional to the severity of the inflammatory status [3,4,11]. Some approaches to the study of the human mycobiome and its origin show that the colonization of the human body by fungi occurs from very early stages of life, when fungi are mainly acquired from the mother, other caregivers, and likely from the environment [12]. Regarding the fungal components of the LRT, it has been considered that they can be inhaled from the environment [7,8] and/or be aspirated from the upper airway, mouth, and nose [13,14,15]. For some opportunistic infections like pneumocystosis, it has been demonstrated that the fungus is acquired from early stages of life and is carried in the lungs of many children [16,17] and other population groups with different percentages of colonization [18]. The development of the opportunistic infection is attributed to the decrease of immune system efficiency [19]. This highlights the importance of knowing as much as possible about the lung mycobiome and the factors that can influence its stability and composition.

The relationship of the lung mycobiota and the environment has been explored in some works, mainly with the aim of ascertaining the connection between environmental mycobiota and the development of allergies. Several culture-based studies have attempted to demonstrate the link between asthmatic crises in children and the presence of fungal components in the air of their homes [20,21,22]. More recently, some studies have approached the same objective through various molecular methods by studying the mycobiome of environments at work [23,24,25]. Most of these studies have shown that the lung mycobiome mainly proceeds from inhaled air and oral cavity mycobiota [26]. Opportunistic mycoses, especially those known as IFIs (Invasive Fungal Infections), are a problem of growing interest due to the recent increase in the number of patients susceptible to them. Some of the fungi most frequently involved in these processes are commonly found in the environment. Therefore, more precise knowledge of the microbiome of the lower respiratory tract and how it is influenced by the microbiota of the environment can constitute an important tool for the prevention of not only allergies but also opportunistic infections. It would contribute to more personalized medicine. These facts led us to perform a study of the relationship between the mycobiome of the lower respiratory tract of adult patients and the mycobiota detected in their domestic environment. More specifically, we focused on an analysis of patients submitted to bronchoscopy due to causes other than infectious diseases since bronchoscopy samples from healthy patients are scarce and Broncho-Alveolar Lavage (BAL) is considered the best sample to explore the microbiome of the LRT [27]. The study is based on molecular (HTS) methods, but cultures were also performed to assess the presence and viability of the fungi detected.

## 2. Materials and Methods

### 2.1. Study Design and Participants

This is an observational study carried out from 2015 to 2019 including 45 patients attending three University Hospitals in the area of the Valencian Autonomous Community (Spain). Selected patients met two important criteria: (1) they permitted a sample of the lower respiratory tract to be taken by bronchoscopy and (2) they were not at risk of suffering lung dysbiosis (at least presumably). The first requirement led us to select patients who had a clinical reason, which justified bronchoscopy. For the second, we had to reject patients under antibiotic treatment, or who had any kind of immunodepression, as these situations can disturb the microbiome and cause a dysbiosis (Supplementary material. Document S1: Patient data sheet). Therefore, the inclusion criteria were: adult patients with an indication for bronchoscopy, not suffering from or showing symptoms of infectious disease and/or immune deficit, and not treated with antibiotics for at least four weeks prior. The study received the approval of the ethics committees of all the hospitals involved (reference codes: PI2017/68, MICROBERESPV4, F-EG-CCI-9) and the University Miguel Hernandez (ref. code: DVM.CVM.01.14). Patients were informed about the objectives of the study and gave their informed consent for the use of their BAL sample. Twenty of them also allowed entrance to their homes for environmental sampling (Supplementary material: Figure S1: Project work flow).

### 2.2. Obtention of Broncho-Alveolar Lavage Samples by Bronchoscopy

Samples were obtained by bronchoscopy with instillation and subsequent aspiration of about 50 mL of sterile saline solution (NaCl 0.9%) [28], which were collected in sterile plastic tubes. Immediately afterward, they were transported to the laboratory at 4 °C in a polystyrene box with cooling blocks. Once in the lab, the samples were divided into 1–1.5 mL aliquots (4–12 aliquots per sample). Four of these aliquots were separated and kept at 4 °C to be immediately processed for conventional culture-based mycology studies and for DNA extraction. The rest were frozen and kept at −80 °C in the Biobank of the General University Hospital of Alicante. Additionally, five of the bronchoscopes were also tested by washing the device with sterile saline before use. The solution was collected and treated like the BAL samples (BS samples).

### 2.3. Environmental Sampling

The addresses of patients were located on a map with the aid of Google Maps software [29] and sampling was scheduled for groups of two or three patients per day, according to the proximity of their houses. Environmental sampling was carried out during six different days in nine cities or towns in an area of about 2000 square km in the Province of Alicante (Southeast of Spain). Once in the house, plates with Potato Dextrose Agar (PDA) were distributed in each of the rooms. Open plates were exposed to the air for at least 10 minutes, while swab samples were also taken in the same rooms. Swabs were previously soaked in saline solution with 0.05 g/L of chloramphenicol and subsequently used to rub the exposed surfaces (furniture, walls, air conditioner openings, sinks, shelves, kitchen countertop, and others). When pets or plants were present in the house, samples directly related to them were also collected (swabs of pet beds, birdcages, plant leaves and stems, bird feces, water from aquariums for fish and turtles, etc.). Swabs were kept inside their cases containing 2 mL of the chloramphenicol solution and other samples were kept in sterile tubes [30]. Data about the house and its environment were recorded during the visit (Table 1 and supplemental material: “patients & houses”). All samples were transported to the laboratory in less than three hours. The number of samples per house ranged between seven and 17 (median: 10). In the laboratory, the exposed PDA plates were kept at room temperature for incubation. Swabs with antibiotic solution and liquid samples were vigorously vortexed and allowed to settle for 15 min. Solid samples were weighed, suspended (approximately 1 gr/10 mL) in the same antibiotic solution, vortexed, and allowed to settle for 15 min. From each supernatant, 100 µL were inoculated in plates of Sabouraud Dextrose Agar (SDA) and PDA and another 100 µL were separated in a microtube for the DNA extraction.

### 2.4. Cultures

For BAL samples, 0.1 mL of each of two aliquots of the sample were inoculated in SDA and PDA plates and subsequently incubated at 37 °C and at room temperature, respectively. The supernatants from the environmental samples were processed in the same way as the BAL samples. All cultures were incubated for four weeks and examined for fungal growth every two or three days. The colonies were isolated in the same media and studied by macro-morphology and micro-morphology and other phenotypic features [31,32] as well as by molecular methods after the extraction of DNA from pure cultures.

### 2.5. DNA Isolation

One aliquot (1 mL) of BAL sample from every patient, together with one combined sample from the domestic environment (ENV sample), was used for the study of the mycobiome. From the samples obtained in the same house, 100 µL of each supernatant were mixed all together, creating a pool which constituted the unique environmental sample (ENV) corresponding to each patient. Each ENV and BAL sample was submitted to DNA extraction by the same procedure.

For DNA extraction, the E.Z.N.A. Yeast DNA kit (Omega Bio-tek Inc., Norcross, GA, USA) was used. The fungal Internal Transcribed Spacer (ITS1-4) was amplified from the extracted DNA by PCR with primers ITS1 (5′ TCC GTA GGT GAA CCT GCG G 3′) and ITS4 (5′ TCC TCC GCT TAT TGA TAT GC 3′) [33,34]. The product was purified by the GeneJet DNA Purification Kit (ThermoFisher Scientific Lithuania, EU) and the concentration of DNA was measured by NanoDrop Lite (ThermoFisher Scientific Inc.). BAL samples had a mean concentration of 79.2 ng/µL with a median value of 64.7 ng/µL (range 15.9–179.7 ng/µL) while, for ENV samples, the mean value was 27.64 ng/µL and the median value was 11.4 ng/µL (range: 6.4–121.7 ng/µL). The fragment of about 300 bp spanned by ITS86 (5′ GTG AAT CAT CGA ATC TTT GAA C 3′) and ITS4 primers inside the barcode region of the ITS2 of fungi was amplified by semi-nested PCR [34] and sequenced by the Solexa system (Illumina, San Diego, CA, USA). The extraction of DNA from a pure culture of isolated fungi was performed mainly by the InstaGeneTM Matrix (Biorad Laboratories Inc., Hercules, CA, USA).

### 2.6. Illumina High-Throughput ITS2 Region Sequencing and Bioinformatic Analyses

Forty-five BAL, 20 ENV, and 5 BS samples, together with a negative PCR control, were submitted to massive sequencing after the first amplification of the ITS-5.8S region (SRA accession number: PRJNA474914). The QIIME 1.8.0 pipeline [35] was used for data processing. Sequences were checked for chimeric sequences with identify_chimeric_seqs.py script against the UNITE database (version 2017-12-01) (https://unite.ut.ee/community.php) using UCHIME [36] Afterward, one negative control from PCR and a negative control from each sequencing run were used to filter contaminants. Samples were clustered in different fungal Operational Taxonomic Units (OTUs) at 99% identity and sequences in OTUs present in control samples were removed for the subsequent analysis. After that, 25 samples (35.7%) were discarded to avoid errors in the analysis due to the low number of sequences [37]. Clean sequences were clustered in OTUs at 97% identity and identified using pick_open_reference_otus.py script. Due to the large difference in library size among samples, the OTU table was rarefied to 6000 reads with a single_rarefaction.py script for comparisons across samples [38] (Supplementary material. Figure S2: Rarefaction curves). Alpha diversity metrics (total observed number of OTUs, and Shannon-Wiener diversity) were generated from the rarefied OTU table using alpha_diversity.py script. Differences among microbial assemblages were assessed with UniFrac analysis, which is a β-diversity measure (differentiation at diversity level among habitats) that uses phylogenetic information using jackknifed UPGMA (weighted pair group method with arithmetic mean) clustering based on the weighted UniFrac (Lozupone and Knight, 2005). These results were plotted in three dimensions with the UniFrac-based principal coordinate analysis (PCoA) [39]. Occasionally, NCBI and EMBL nucleotide databases (BLASTn update 2019) were also used for identification of highly prevalent OTUs that could not be assigned to a species or genus by the UNITE database.

## 3. Results

### 3.1. Characteristics of Participants

**Figure 1:**
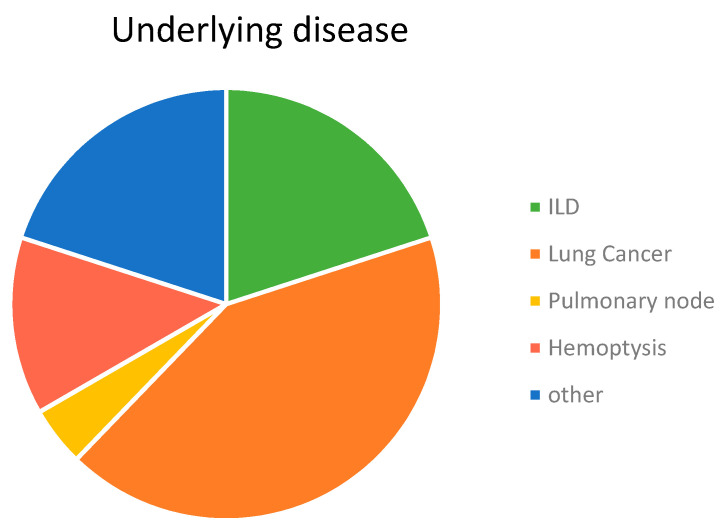
Distribution of patients according to the clinical situation that indicated the bronchoscopy. Others included: Wegener syndrome, dysnea, thymoma, TBC sequels, atelectasis, lung infiltrate, tracheal stenosis, Hodking and and Non-Hodgkin lymphoma, COPD, and cough.

Demographic characteristics of the patients and their houses are shown in Table 1. The studied population consisted of 25 male (55.6%) and 20 female (44.4%) patients. Bronchoscopy was indicated on the basis of 15 different non-infectious clinical situations with lung cancer being the most frequent (42.2%), which was followed by diffuse interstitial lung disease (ILD) (20%), hemoptysis (13.3%), and pulmonary nodules (4.4%). Eleven additional pathologies were detected including one in each of eleven patients (Figure 1). The professional activity of the patients was quite varied in which the largest group was related to the footwear industry (20%), known as one of the most important in the area, which was followed by building (15.5%). Nine patients (20%) declared that they had had more than one profession throughout their lives. With regard to foreign travel, almost half of the patients (22) had never left Spain and among the others, 13 (28.9%) had only visited one country/city. Nine patients (22.2%) declared they had travelled to different destinations in the world. Finally, 33.3% of the patients declared having had exposure to animals or animal products. Most of them (6/15) had birds in cages (canaries, a goldfinch, and a siskin) and 40% (6/15) had pigeons, hens, and/or quails. Of the 20 patients who allowed sampling of their homes, 13 (65%) lived in flats in an urban environment and seven (35%) lived in houses located in villages (2), on town limits (3), or in rural areas (2). Pets were present in seven (35%) of the studied dwellings (see Supplementary Materials. Table S1: patients and houses).

### 3.2. HTS-Based Mycobiome Characterization

#### 3.2.1. Richness and Diversity

**Figure 2:**
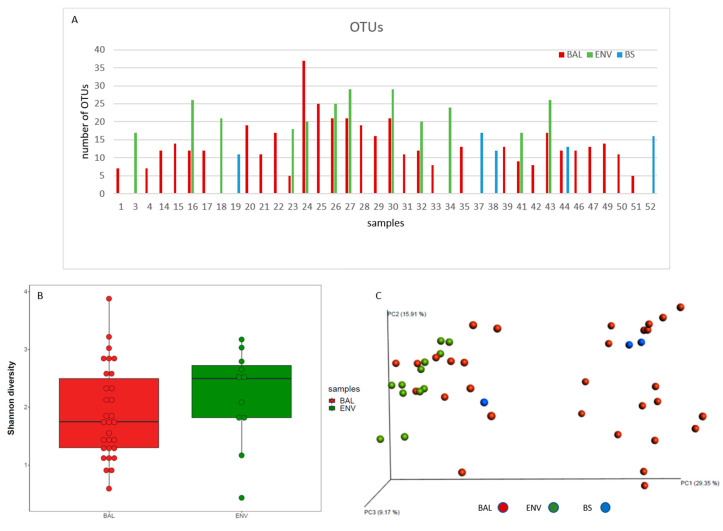
Fungal diversity in the studied simples. (**A**) Representation of the number of different OTUs detected in each sample. (**B**) Shannon-Wiener value for BAL and ENV samples. (**C**) Beta diversity. Bray-Curtis dissimilarity coefficient scores PCoA.

Forty-five BAL, 5 BS, and 20 ENV samples submitted to massive sequencing analysis yielded 7,015,877 sequences (5,046,424 from BAL, 1,861,275 from ENV samples, and 108,178 from BS). After chimeric detection and contaminant filtering, 4,012,543 ITS sequences were included in the subsequent analysis (2,826,988, 1,110,227, and 75,328 from BAL, ENV, and BS samples, respectively) with a mean value around 60,000 for BAL and ENV samples and 15,000 for bronchoscopes (Table 2). After sample rarefaction, 77 fungal Operational Taxonomic Units (OTUs) at 97% identity were identified, ranging from 5 to 37 in BAL samples, from 17 to 29 in ENV samples, and from 11 to 17 in BS samples (Figure 2A). Shannon-Wiener index ranged from 0.6 to 3.9 in BAL samples and from 0.4 to 3.2 in ENV samples (Figure 2B, Table 3). The similarity among different microbial communities (beta diversity) analysed by the Bray-Curtis index showed a very high similarity among domestic environmental samples (average 21.88) and a high diversity in BAL samples (average similarity 9.93). Accordingly, all ENV samples clustered together while BAL and BS samples were distributed in two different clusters (Figure 2C).

#### 3.2.2. Taxonomy

**Figure 3:**
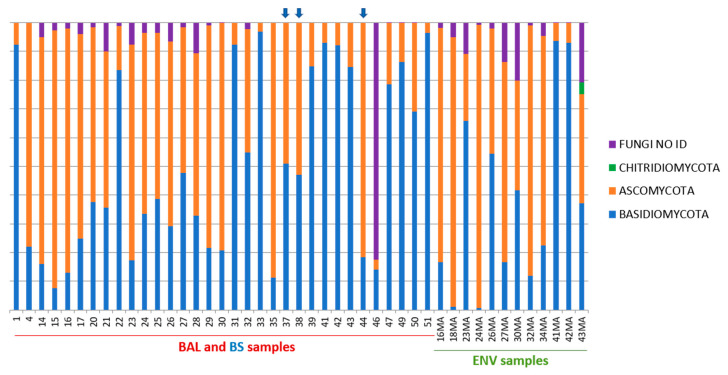
Distribution of OTUs at a phylum level in each BAL, BS, and ENV samples. Upper blue arrows point at BS samples.

*Basidiomycota* and *Ascomycota* phyla were detected in all types of samples with a relatively higher presence of *Basidiomycota* in the lungs (BAL samples 49.4%) than in environmental samples (38.2% in ENV) that had a slightly higher percentage of *Ascomycota* (54.65% in ENV) (Figure 3). The phylum *Chytridiomycota* was very poorly represented and only detected as 0.33% of the sequences in ENV samples (Table 3 and Figure 3). Bronchoscopes were shown to be contaminated by some of the fungi detected in lungs even though the number of sequences in these samples was very low (Table 2).

In our study, 1.1% of the detected sequences could belong to new species or genera as they could not be identified below family level (Supplementary material. Table S2: Taxonomy). These corresponded to 13 OTUs (16.9 %) that remained identified at different taxonomic levels. One of these OTUs (OTU29) identified as “unknown fungi” had no match under the kingdom level and was detected in many BAL and ENV samples. Other OTUs could only be classified at phylum *Ascomycota* (3.9%) or at different orders level (10.4%), according to the UNITE database (Table 4).

#### 3.2.3. Core Mycobiome

**Figure 4:**
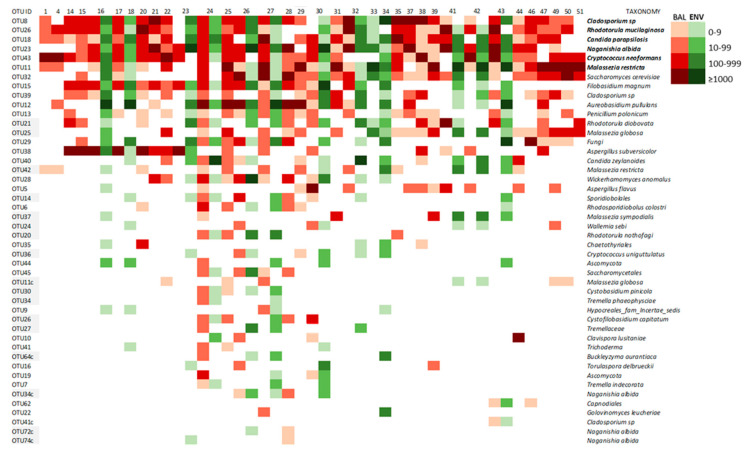
Global distribution of putative fungal species detected in BAL, ENV, and BS samples. The intensity of the color corresponds with the number of sequences in a logarithmic scale. The left column shows the identification number of each specific OTU and their correspondent species are in the right column.

**Figure 5:**
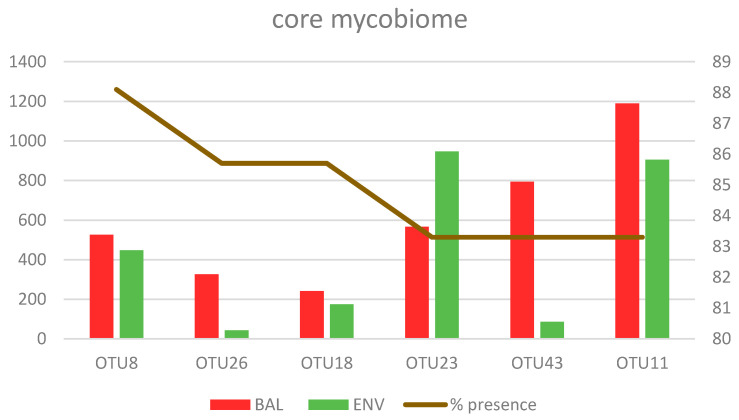
Presence of the six most prevalent OTUs in BAL and ENV samples. The relative presence is represented by the percentage of samples in which they were detected and the abundance as the mean value of sequences per sample.

OTUs that were present with sufficient relative abundance in more than 85% of the samples were considered a core mycobiome. For BAL samples, OTU8 that was present in 90.9% of samples. OTU26 and OTU43 were both present in 86.7% of them and were considered as the core mycobiome of the lungs. These three OTUs presented hits with *Cladosporium* sp., *Rhodotorula mucilaginosa*, and *Cryptococcus neoformans*, respectively (Figure 4). For the environmental samples, five OTUs (8, 12, 15, 18, and 23) that matched with *Cladosporium* sp., *Aureobasidium pullulans*, *Filobasidium magnum*, *Candida parapsilosis*, and *Naganishia diffuens*, respectively, were detected in 100% of the ENV samples studied and, therefore, considered as the core mycobiome of the houses. Five of these OTUs have an important presence in both kinds of samples from patients and environments (Table 5). Additionally, the OTU11 that matched with the species *Malassezia restricta* was detected in 83.3% of lungs and houses and was equally highly abundant in both of them (BAL and ENV) (Table 5 and Figure 5).

#### 3.2.4. Lung Mycobiome

**Figure 6:**
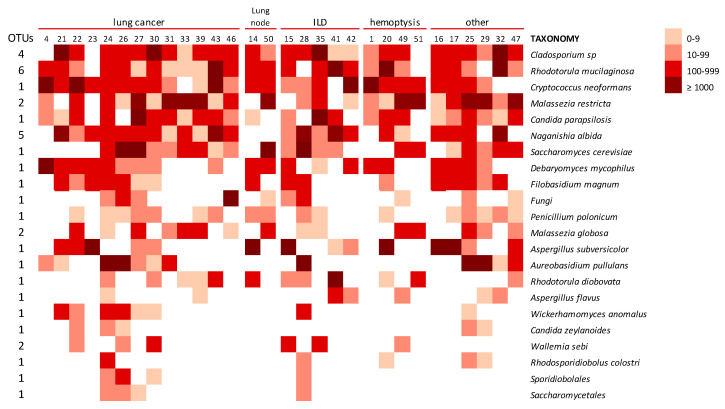
Distribution of fungal species detected in BAL samples clustered by the underlying disease or symptoms (others corresponded to: Thymoma, TBC sequels, Atelectasis, lung infiltrate, tracheal stenosis, and COPD, respectively). The intensity of the color corresponds with the number of sequences that match with the putative species in the UNITE database at 97% similarity (logarithmic scale). The column at the left identifies the number of OTUs that showed a hit with the same species shown in the left column. Species present in less than 10% of the samples and with less than 100 sequences are not shown.

The fungal lineages constituting the BAL core mycobiome look very highly micro-diverse, as indicated by the presence of several OTUs being less abundant than the ones in Figure 5 but still matching to the same species. The number of these additional OTUs for *Cladosporium* sp. and *Rh. mucilaginosa* was four and six, respectively. These results reinforce the important presence of these fungi in the lungs. Other taxa with several OTUs detected in BAL samples corresponded to *N. albida* (5 OTUs), *M. restricta* (2 OTUs), *Malassezia globose* (2 OTUs), and *Wallemia sebi* (2 OTUs) (Figure 6). The distribution of different taxa was heterogeneous among the patients with different symptoms and diseases, and every analyzed individual had a specific mycobiome profile, which could not be related to the diagnosis nor clinical status (Figure 6).

#### 3.2.5. The Environmental Mycobiome

**Figure 7:**
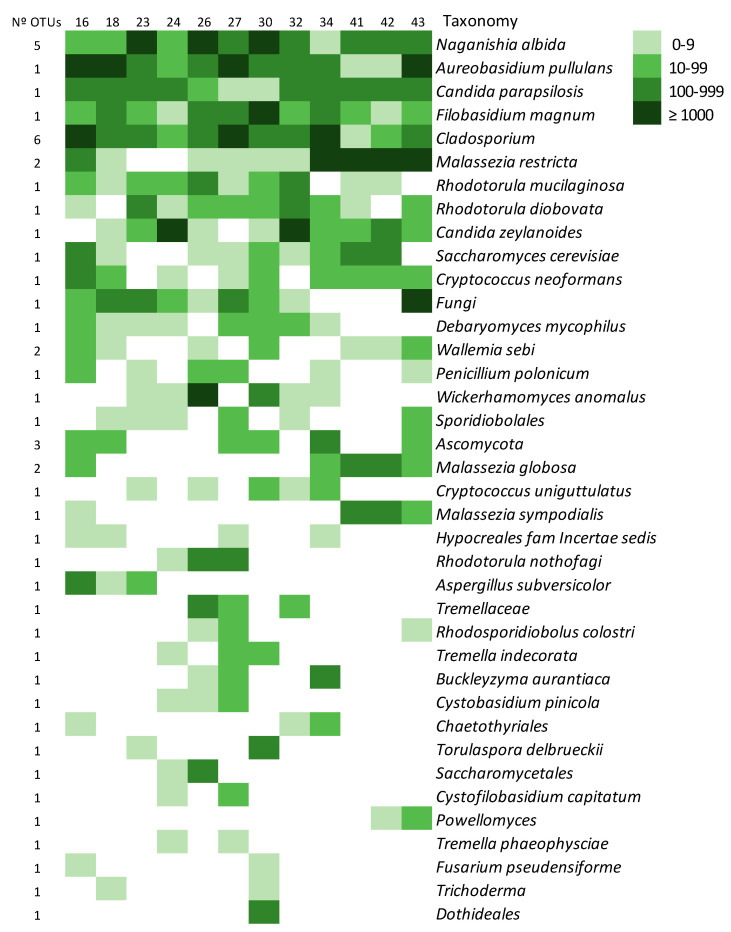
Distribution of fungal putative species detected in ENV samples. The intensity of the color corresponds with the number of sequences that showed a hit with the species in the UNITE database at 97% similarity (logarithmic scale). The column at the left shows the number of OTUs that corresponded to each of the putative species. The ones present in less than 10% of the samples and with less than 100 sequences are not shown.

In samples from houses (ENV), three species were detected in all of them: *Naganishia albida, Aureobasidium pullulans, Filobasidium magnum, Cladosporium* sp., and *Candida parapsilosis*, which had 947, 1323, 565, 191, and 174 sequences per sample, respectively. These species constitute the core mycobiome of the domestic environment (Figure 7). Other species like *Malassezia restricta*, *Rh. mucilaginosa, Rh. diobovata*, and *Candida zeylanoides* were present in 83.3% of the samples with a high number of sequences. As with the lungs, each domestic environment had its specific profile, which was not related to the type of house, area of the location, or presence of pets (Figure 7).

#### 3.2.6. Mycobiome of Lungs and Environments

**Figure 8:**
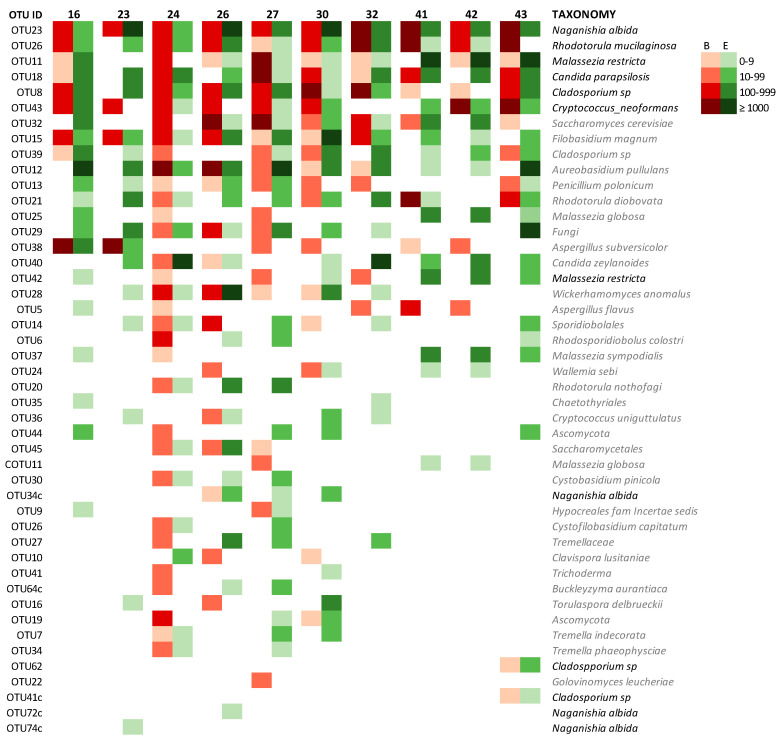
Comparison between lung and environmental mycobiome. BAL and ENV profile of OTUs of each patient who permitted the analysis of both samples. The intensity of the color corresponds with the number of sequences that match with the species in the UNITE database at 97% (logarithmic scale). The column at the left shows the identification number of the OTU. The correspondent species are in the right column.

The comparison of the lung and the house mycobiome of the same patient is represented on the heatmap of Figure 8. Results obtained from BAL and ENV of patients that offered the two kinds of samples were analyzed and the similarity of the two profiles (lung and house) for each patient was assessed as the percentage of coincidence in the presence/absence of each OTU in both environments. The coincidence ranged from 71.4% to 81.8% with a mean value around 75% (Table 6). In general, lungs and houses mostly shared the OTUs corresponding to the fungi described as core mycobiome. In patients 16, 27, 30, and 32, these OTUs are 100% coincident (Figure 8). The similarity is also demonstrated by the presence of minority fungal OTUs like the one corresponding to *A. subversicolor* (OTU38), which is present in BAL and ENV of patients 16 and 23, or OTU21 (*Rh. diobovata*) coincident in BAL and ENV of patients 24, 30, 41, and 43 and the very rare OTUs 62 and 41, that were only detected in patient 43 in both samples BAL and ENV (Figure 8). The similarity of the fungal profiles is more evident for patients whose samples had higher fungal richness. In this regard, patients 24, 26, and 30 had mycobiomes highly diverse in the number of different OTUs that also were highly abundant in sequences (Table 6) and these three patients show around 50% of coincidence in the OTUs present in their two samples and from 75.3% to 79.2% of coincidence in the global profile. The situation of OTU12, which corresponds to *A. pullulans*, is of interest. This fungus had been described as highly prevalent in indoor samples [40] and we detected it in all ENV samples studied, and also in 43.3% of the BAL samples and in one bronchoscope (33.3%). The presence/absence of this species (*A. pullulans*) was the only variable that could explain the distribution of BAL and BS samples into two different clusters by the Bray-Curtis index (Figure 2C).

### 3.3. The Culturable Mycobiome

Cultures of BAL samples were mainly negative. However, after the incubation period, one or a few fungal colonies were detected in 46.7% (21) of the plates. The phenotypic identification of these fungi showed species of the genus *Rhodotorula* in the samples of five patients: *Penicillium* spp. and *Aspergillus* spp. in the BAL of four and three patients, respectively, *Naganishia* sp. in three, *Cryptococcus neoformans sensu lato* in two of the samples, and *Candida* sp and *Cystobasidium* sp. one of each in the sample of the same patient (patient 3). The molecular identification of some of these colonies identified them as *Rh. mucilaginosa*, *P. glabrum*, and *Cr. neoformans sensu stricto*. Most of the fungi isolated in cultures were also detected in the HTS study of the same patients. Samples 4 and 41 had *N. albida* in their HTS study and their cultures showed one CFU of a morphotype of *Naganishia* spp. in each sample. *Rh. mucilaginosa* was detected by HTS in the lungs of patients 33 and 37 and their cultures shown one and six CFU, respectively, of a yeast with morphotype corresponding to *Rhodotorula* sp. The culture of the BAL sample of patient 22 had two filamentous colonies corresponding with morphotypes of *Penicillium* spp. and *Aspergillus* spp. and the HTS analysis showed the presence of *Penicillium polonicum* and *Aspergillus subversicolor* in these samples. Finally, from the BAL sample of patient 23, *P. polonicum* was also identified by massive sequencing and a colony of a fungus morphologically corresponding to *Penicillium* spp. was obtained. Other isolates were obtained from cultures of BAL samples that were discarded for the HTS analysis after the procedure of cleaning the sequences and, therefore, we cannot compare the two methods. This is the case of patient 3 in whose sample the growth of two CFU corresponding to *Candida* sp., two to *Naganishia* sp., and one to *Cystobasidium* sp. were detected (Supplementary materials. Table S3: cultures).

**Figure 9:**
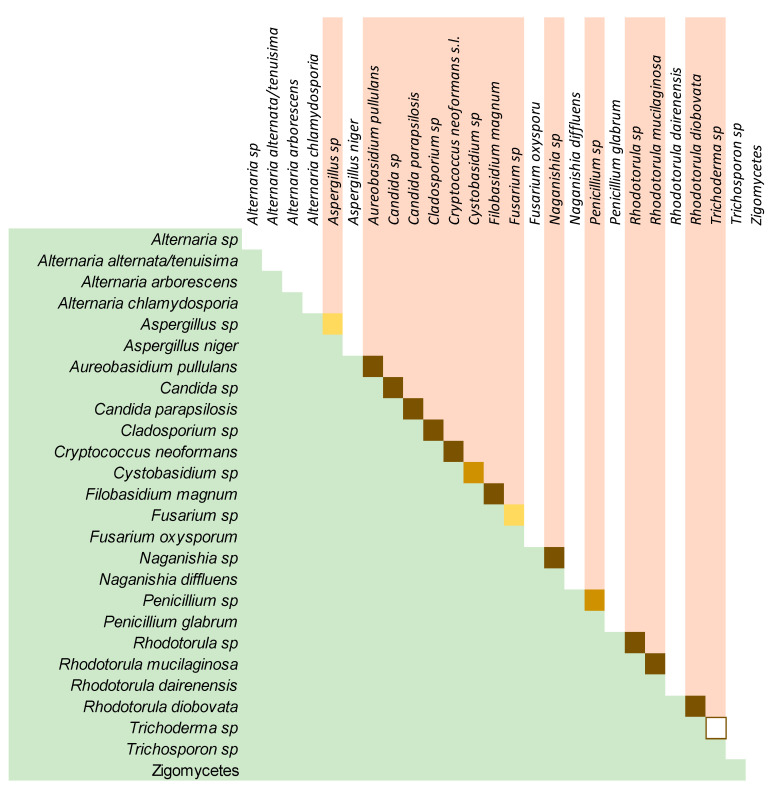
Concordance between the detection of fungi by culture and by the HTS method. Green background: Genera and species obtained by the culture. Pink background: same genera and species in the HTS analysis. The co-occurrence of detection in the same samples is represented by squares at the intersection. Dark squares: 100% of positive samples by culture were detected by HTS. Golden squares: 40% and 70% of coincidence in culture and HTS (from left to right and up to down). Yellow squares: from 17% to 25% of coincidence. Empty squares: Detection in different samples (0% coincidence).

In contrast with the BAL samples, cultures of environmental samples produced larger numbers of fungal colonies. Growth was detected in almost all the inoculated plates and a total of 12,994 CFU were detected. These included 387 morphotypes (188 filamentous fungi and 199 yeast-like isolates). Two colonies of each morphotype were selected for further study, except in cases in which only one colony of a specific morphotype was available. That selection gave 718 isolates that were submitted to phenotypical and molecular procedures for the identification at genus or species level and 168 of which were successfully identified (43.4%). The most represented genera in cultures for the yeast-like colonies were *Rhodotorula*, isolated from 80% of the houses, followed by *Trichosporon*, *Naganishia*, and *Candida* in 40% to 55% of houses, *Filobasidium* in only 10%, and *Cryptococcus* in 25% of the 20 domestic environments but with the highest number of colonies in the plates (1340 CFU). Among the filamentous fungi, *Aureobasidium* was isolated in 70% of the houses, and *Penicillium*, *Aspergillus*, *Alternaria*, and *Cladosporium* were present in 40% to 60% of the 20 houses and *Cladosporium* showed the highest number of colonies detected (Supplemental material cultures). All these genera were also detected by HTS in the same samples except for *Trichosporon*, *Alternaria*, and *Aspergillus*. For *Aspergillus*, the phenotypic characterization of the isolates obtained in ENV samples of patients 26, 27, and 30 showed *A. niger* and, in samples 27 and 30, an additional unidentified species of *Aspergillus* was isolated. The HTS analysis did not detect *A. niger* in any of the studied samples and the OTUs corresponding to this genus (OTU5 and 38) were not detected in the houses of these three patients. For *Trichosporon* and *Alternaria*, although both were isolated from almost half of the ENV samples (55% and 45%, respectively), the HTS did not detect these genera. For the genus *Cladosporium*, it was detected in all ENV samples by HTS and cultured from 40% (eight houses) of them (Figure 9).

## 4. Discussion

The composition of the indoor environment is presumably strongly influenced by the air and dust outdoors [41]. Accordingly, Ascomycota have been described as highly predominant in outdoor and indoor environments by previous culture-based studies [20,21,22,42]. In these studies, the house environment is mainly explored in relation to asthma patients and the analysis of fungal components in the environment is mostly focused in the detection of fungi, which are potently allergenic, such as the genera *Aspergillus*, *Cladosporium*, *Penicillium*, or *Alternaria*. Most of these belong to the phylum Ascomycota. In these works, yeasts were almost absent or may not be reported as they are not among the allergenic fungi. Therefore, the phylum Basidiomycota, which harbors many important human-related yeasts, was a rare minority in most of these studies. However, our results, together with other recent molecular studies, show a more significant presence of members of Basidiomycota and describe yeast of the genus *Cryptococcus* and *Malassezia* as highly present in indoor environments [23,24,40,43]. The work of Green et al. [23] specifically described 41% of the fungal microbiota inside a building as members of Basidiomycota and 55% of Ascomycota species, which is almost the same distribution detected in the houses of our patients.

The lung mycobiome. The analysis of the results of the HTS study of the BAL samples offered 30 different profiles in fungal composition. That means each patient had a specific distribution of fungal OTUs in their lung mycobiome (Figure 6), which is in agreement with the findings of other authors [8,44]. Nevertheless, the group of OTUs considered as a core mycobiome were repeatedly detected in these samples and the presence of genus like *Malassezia, Candida*, and *Cryptococcus* coincide with previous reports [11,13,14,45,46,47,48]. However, in these studies, a major presence of *Candida* spp. is described and *Rhodotorula* species were rarely reported previously in the lung mycobiome. The predominance of *Candida* spp., including *C. albicans*, which is absent in our study, would be explained by the frequent use of sputum as the sample representative of LRT mycobiome [7,11,47,49]. That kind of sample would carry fungi from the upper respiratory tract, where *Candida* species are highly prevalent [13,27,50]. Moreover, these studies mainly aim to correlate the mycobiome composition of the lungs with the clinical status of patients suffering from chronic inflammatory pulmonary diseases, such as CF, COPD, asthma, and bronchiectasis [11,49,51,52,53]. Although no specific correlation has been established between disease and mycobiome composition so far, the severity of the inflammatory process seems to have an inverse relationship to the diversity of the microbiome [3,4,8,11]. According to what is described as inflammatory diseases of the lung, such as pneumonia, acute respiratory distress syndrome (ARDS), asthma, and chronic obstructive pulmonary disease (COPD) [54], most of our patients had non-inflammatory disorders (Figure 1). Thus, inflammatory processes may correspond to a more similar mycobiome, less diverse than one of the persons with a “non-inflammatory” status. Among our patients, no significant relationship could be established between fungal profiles and any of their underlying pathologies or clinical situations. However, the high number of sequences of *Malassezia* species in cancer patients should be pointed out (Figure 6).

Apart from the underlying disease, the possible influence of clinical and demographic variables such as age, sex, profession, travel history, type of house, and lifestyle did not show any significant relationship with the richness, diversity, or fungal composition of the BAL samples of these groups of patients.

Relationship between lungs and dwellings. Comparing OTUs instead of species or higher taxa gives higher sensitivity to the analysis. The profiles of samples of the 10 patients that had the two kinds of samples showed that all lungs and houses harbor OTU23, which corresponds to *Naganishia albida*, a fungus that was not frequently described previously as a member of mycobiome. It was a former member of the genus *Cryptococcus* (*Cr. albidus* = *N. albida*) [54] and has been reported in the environment and also as an infrequent opportunistic pathogen [25,55]. Perhaps its presence in other studies is somehow hidden under the *Cryptococcus* spp. Group, which is described in different works [13,23,24,40].

The high similarity of the fungal composition of the LRT of our patients and their dwellings is in agreement with the hypothesis that the environment is one of the most significant determinants of the composition of the human microbiome [9]. Previous culture-based studies showed a higher prevalence of *Aspergillus* and *Penicillium* [20,21,22,42], which are traditionally the most expected genus in indoors environments. It seems that this is a bias of culture-based procedures as our results are coincident with other molecular approaches [24,43,56]. Nevertheless, *Penicillium polonicum* and two species of *Aspergillus* (*A. subversicolor* and *A. flavus*) were detected in 50% (6/12) and 33.3% (4/12) of the samples from houses and the genera were also cultured. Surprisingly, these two genera were more prevalent among the samples from the lower respiratory tract of the patients than from their environment (Figure 8).

Some authors have attempted to establish a difference between what is considered real human microbiota, and the microorganisms that can be detected in the lungs as a result of a transient colonization that most likely come from the environment [57,58]. This distinction is not easy to establish. Although, in our results, the lungs and houses showed different members of the core mycobiome. The species related to the most abundant and prevalent OTUs in the two kinds of samples tend to overlap and there is not enough evidence to declare which ones had evolved to be adapted to the lungs in contrast with the ones that are frequently inhaled and belong to the external environment. The study by Kramer et al. [57] concluded that the mycobiome is dominated by transient species from inhaled air rather than colonization. This situation is estimated by the analysis of sequential sputum samples from patients of cystic fibrosis that were frequently under antibiotic treatment. Sputum carries many microorganisms from the upper area via mouth, and these areas are more exposed to the external environment than the LRT. Therefore, presumably, these samples would reflect a higher variability due to external influence than the BAL ones. Considering that this situation can be extrapolated to the lower area via only speculation. Neither the previously mentioned works nor ours present enough evidence to make a clear distinction between the resident and transient biota of the lower airway.

Cultures confirmed the presence of most of the fungi that were prevalent in the samples studied by HTS. The three species detected as environmental core mycobiome in the molecular study were also detected by culture (*A. pullulans, C. parapsilosis* and *Naganishia* sp.). Other species and genera with a significant presence in the molecular approach like *Rhodotorula* and *Cryptococcus* and the filamentous fungi *Aspergillus, Penicillium*, and *Cladosporium* were also cultured from many environmental samples but also from some of the BAL samples in which *Candida* spp. was also detected. Only the genus *Malassezia*, which had a high relative presence in most of the lungs and houses studied, was not detected by culture, which can be explained by the special requirements of the members of this genus for in vitro growth [59]. The scarce fungal growth obtained from BAL samples compared to one of the same species from ENV samples leads us to consider the vital status of the fungi detected by HTS methods. The very low rate of isolation of fungi whose DNA is detected and that can easily grow in common media led us to conclude that most of them are not viable and they may not even be cells but rather debris of nucleic acids partially destroyed by the local immune system.

The high rate of detection of unidentified fungal sequences seems to be a common fact and has also been reported in other works [41,43,60]. We consider this a significant target for future studies.

## 5. Conclusions

Indoor environmental mycobiome is the result of the mixed outdoor fungal components and human (and pet) mycobiome. Humans exchange fungi with their environment and the proportions of different species or subspecies (OTUs) in both sites are the result of specific factors that the fungi find in each of these environments (temperature, nutrients, pH, immune response, and others). Therefore, some fungi are more prevalent in lungs than in the environment, like *Rh. mucilaginosa, Cr. neformans*, or *Aspergillus* spp., while others are more abundant in the air or dust of houses (e.g., *M. restricta* and *Capnodiales*). In any case, it seems that there is a reciprocal influence of the two environments and fungi inhaled from indoor air contribute to the composition of the human mycobiome and, in the same way, the human mycobiome may influence the indoor environmental mycobiota, which also receives most of its members from the outdoors.

Fungal species, such as *Rh. mucilaginosa, C*. *parapsilosis*, *N. albida*, *M. restricta*, *A. pullulans*, *Cr. neoformans sl.*, and *Cladosporium* spp., are the most prevalent in both environments studied. This agrees with previous studies but also adds more information about species that were less frequently described, such as *Naganishia* spp. Nevertheless, we have to assume that the study of a unique molecular target obviates the presence of fungi-like *Pneumocystis jirovecii*, which is a well-known commensal of the LRT of different groups of patients and the normal population [18] but cannot be detected by this approach due to its low number of copies of the ITS region [61]. Perhaps other unknown fungi may remain hidden in the mycobiome when the study is only based on ITS amplicon sequencing. In addition, in this study as well as in many others performed with the same molecular approach, a significant number of OTUs could not be related to any specific fungal taxon through the UNITE database and other databases. That means that much remains to be explored regarding the lung mycobiome and the house environment, and their role in human health and disease.

Our results confirm that the environment could be the main determinant of the lung mycobiome, and that the health condition of individuals, specifically their inflammatory status, may influence the diversity and richness of the mycobiota more than its composition. However, it is important to note that mycobiome analysis offers only a partial view of the state of the microbiome of an environment, and its relationship to other microbial communities (bacteria, protozoa, viruses, and archaea) should always be considered for a robust global assessment.

## Figures and Tables

**Table 1:** Patient data. Summary of demographic data of patients (complete information is submitted as supplementary material Table S1).

AGE RANGE		PROFESSION	
40–49	2	Shoes industry	9
50–59	15	Building	7
60–69	13	Administrative	4
70–79	10	House wife	3
80–89	5	Grocery	4
SEX	25 MEN (55.6%)	Other (less than 2 each)	20

**Table 2:** Number of sequences by type of sample before and after cleaning.

Sample	Total Before Cleaning	Total Clean	Mean/Sample
BAL	5,046,424	2,826,988	62,822
ENV	1,861,275	1,110,227	58,433
BS	108,178	75,328	15,066
TOTAL	7,015,877	4,012,543	

**Table 3:** Distribution of sequences of the three classes of fungi and alpha diversity (mean value of Shannon-Wiener index) by the type of sample after rarefaction.

*Fungal Class*	BAL			ENV			BS		
	Total	Mean	%	Total	Mean	%	Total	Mean	%
ASCOMYCETES	85,242	2750	45.85	39,348	3279	54.65	5256	2628	43.8
BASIDIOMYCETES	91,948	2966	49.44	27,521	2293	38.22	6744	3372	56.2
CHYTRIDIOMYCETES	0	0	0	247	20.6	0.33	0	0	0
FUNGI UNIDENTIFIED	8774	283	4.7	4884	407	6.8	0	0	0
TOTAL	185,964		99.99	72,000		100	12,000		100
alpha diversity	1.901600765			2.144806055			1.693532242		

**Table 4:** OTUs that did not produce hits identified at a genus level by 97% similarity in the UNITE database.

OTU ID	Taxonomy UNITE	LBA			ENV		
		Samples	%	SEQ	Samples	%	SEQ
OTU29	*Fungi*	10	32.3	5470	10	83.3	2588
OTU19	*Fungi; Ascomycota*	2	6.4	301	2	16.7	15
OTU44	*Fungi; Ascomycota*	1	3.2	34	5	41.7	228
OTU35	*Fungi; Ascomycota; Eurotiomycetes; Chaetothyriales*	2	6.4	130	3	25	76
OTU45	*Fungi; Ascomycota; Saccharomycetes; Saccharomycetales*	4	12.9	131	2	16.7	351
OTU14	*Fungi; Basidiomycota; Microbotryomycetes; Sporidiobolales*	4	12.9	257	6	50	60
OTU27	*Fungi; Basidiomycota; Tremellomycetes; Tremellales; Tremellaceae*	1	3.2	64	3	25	283
OTU9	*Fungi; Ascomycota; Sordariomycetes; Hypocreales; Hypocreales fam Incertae sedis*	1	3.2	75	4	33.3	9

**Table 5:** OTUs that were the most prevalent in both kinds of samples. Percentage of samples in which they were detected and abundance in the number of sequences.

OTU N°	PRESENCE IN SAMPLES (%)			N° OF SEQUENCES (Mean Value)			Taxonomy
	BAL	MA	TOTAL	BAL	MA	TOTAL	
OTU8	90.9	83.3	88.10%	526.3	447	563	*Cladosporium* spp.
OTU26	86.7	83.3	85.70%	326.3	43.3	244	*Rhodotorula mucilaginosa*
OTU43	86.7	75	83.30%	794.5	85.6	611	*Cryptococcus neoformans*
OTU18	80	100	85.70%	241.6	174.4	224	*Candida parapsilosis*
OTU23	76.7	100	83.30%	566.8	947.4	650	*Naganishia albida*
OTU11	83.3	83.3	83.30%	1189.1	905	1023	*Malassezia restricta*

**Table 6:** Degree of co-occurrence of OTUs in the mycobiome of the lung and the house of 10 patients who offered both kinds of samples.

PATIENT ID	16	23	24	26	27	30	32	41	42	43
coincident OTUs (present + absent)	61	63	58	61	55	61	63	61	60	56
% coincidence (present + absent OTUs)	79.2%	81.8%	75.3%	79.2%	71.4%	79.2%	81.8%	79.2%	77.9%	72.7%
OTUs present	27	19	38	31	36	33	23	21	21	32
Total sequences (BAL + ENV)	141,606	183,668	761,132	214,953	85,412	234,499	140,075	34,002	17,158	83,962
coincident OTUs (present)	11	4	19	15	14	17	9	5	4	11
% coincidence (present OTUs)	40.7%	21.1%	46.2%	48.4%	38.9%	51.5%	39.1%	23.8%	19%	34.4%

## Supplementary Materials

The following are available online at https://www.mdpi.com/2076-2607/8/11/1717/s1. Table S1: patients & houses. Table S2: taxonomy of the identified OTUs. Table S3: cultures. Figure S1: Project work flow. Figure S2: Rarefaction curves. Document S1: patient data sheet.
