# Supplementary material for: The Domestic Environment and the Lung Mycobiome
Source: Microorganisms. 2020 Nov 2;8(11):1717. doi: 10.3390/microorganisms8111717 (PMC7693370; doi:10.3390/microorganisms8111717)
Supplement: Supplementary file 1 [file microorganisms-08-01717-s001.pdf]

Table S1. Patients included in the study. Clinical and demographic data and variables related to their houses

LBA SAMPLE	SEX	AGE	ENVIRONMENTAL SAMPLE	UNDERLYING DISEASE	PROFESSION	TRAVELS	AREA (rural/urban)	TYPE OF HOUSE	PETS
**1**	F	40		Hemoptysis	shoe maker	No			No
**2**	M	53		ILD	warehouse forklift driver	No			No
**3**	M	60	**3ENV**	cancer (neuroendocrine)	Grocery/administrative in metal factory	No	Limit urban/rural	house	fi sh
**4**	M	59		epidermoid carcinoma	Shoe industry (leather warehouse)	Portugal 2012			No
**5**	M	48		Wegener syndrome	Shoe maker	No			No
**6**	M	64	**6ENV**	ILD	Building (retired)	No	urban	flat	No
**7**	M	64	**7ENV**	Hemoptysis/bronchiectasis	concierge	France, England and Ireland 2005 Portugal 2007	urban	flat	canary
**9**	M	73	**9ENV**	oat-cell cancer	Shoe industry	No	urban	flat	No
**10**	M	41	**10ENV**	ILD/systemic sclerosis / adenocarcinoma	Draughtsman (architecture) Unemployed	England 2013	urban	flat	No
**11**	M	59	**11ENV**	Hemoptysis	locksmith	No	urban	flat	No
**12**	F	68	**12ENV**	Dyspnea / Relapsing Polychondritis	canning industry worker	Prague 2000	urban	flat and shop	No
**14**	F	77		Pulmonar node	Cleaning service (Kindergarden)	France			No
**15**	M	58		ILD	Transport and hostelry	No			No
**16**	F	53	**16ENV**	Thymoma	grocery	No	urban	flat	turtles
**17**	M	72		TBC sequels	builder	Rome, Monaco, Istambul			No
**18**	M	65	**18ENV**	Hypersensitivity pneumonitis	show manager	Frequently: Eastern Europe, SouthAmerica and Cuba. Prague 2016; Portugal 2017	Limit urban/rural	house	NO
**19**			**CONTROL BCP**						
**20**	M	71		Hemoptysis	Shoe industry (leather warehouse)	No			hens
**21**	M	68		adenocarcinoma	Car seller; hostelry	France			canaries
**22**	M	68		adenocarcinoma	Builder, agriculture, hostelry	No			Pigeons (in the proximity)
**23**	M	55	**23ENV**	epidermoid carcinoma	Shoe industry (leather craftsman)	Melilla (North Africa)	urban	flat	No
**24**	M	74	**24ENV**	epidermoid carcinoma	Plumber	Bordeaux	urban	flat	No
**25**	F	81		Atelectasis	Shoe maker, warehouse, grape harvester	No			No
**26**	M	73	**26ENV**	epidermoid carcinoma	Builder	No	limit rural/urban	house	hens, quails
**27**	M	76	**27ENV**	oat-cell cancer	Notary officer	rome, Monaco, Istambul	rural	house	No
**28**	F	84		ILD	Shoe industry	Greece, Hungary, Czech Republic			No
**29**	F	56		right lung infiltrate	Administrative	France			No
**30**	F	73	**30ENV**	metastasis (kidney tumor)	Baker; Builder	No	rural	farm	Siskins, canaries, pigeons
**31**	M	55		microcitic cancer	Builder; Real estate agency	Africa			No
**32**	F	63	**32ENV**	Tracheal stenosis	Bakery; Sausage factory	Indonesia, Argentina, Dominican Republic, Morocco	urban	house	goldfinch, canaries
**33**	M	57		oat-cell cancer	gardener	Bordeaux			canaries
**34**	F	58	**34ENV**	ILD	Cleaning service (Kindergarden)	No	limit rural/urban	flat	tortoises
**35**	M	61		ILD	Builder	No			canary
**36**	F	70		Hodking lymphoma	Retired	No			pigeons
**37**			**CONTROL BCP**						
**38**			**CONTROL BCP**						
**39**	F	56		adenocarcinoma	Housewife	No			No
**40**	F	84		metastasis (gastric tumor)	Cleaning service	France			No
**41**	M	70	**41ENV**	ILD	Administrative	Czech Republic	urban	flat	No
**42**	M	69	**42ENV**	ILD	telephone pole installer	Sweden, France	urban	flat	No
**43**	F	62	**43ENV**	adenocarcinoma	Chemist. University Professor	Cuba, Ecuador, France	urban	flat	no
**44**			**CONTROL BCP**						
**45**	F	52		No Hodking Lymphoma	Administrative	No			No
**46**	F	85		adenocarcinoma	Housewife	No			No
**47**	F	57		COPD	Housewife	USA; Holand			No
**48**	F	54		cought	caregiver children (school)	no			dog
**49**	F	56		Hemoptysis	elderly caregiver	Italy			No
**50**	M	64	**50ENV**	Pulmonar node	Butcher	Jerusalem, Gaza, Morocco, Egypt, Sahara, Palestine	urban	house	No
**51**	F	65		Hemoptysis	Shoe maker/Housewife	NO			Pigeons (in the vicinity)
**52**			**CONTROL BCP**						
**53**			**CONTROL PCR**						
ILD	DIFFUSE INTERSTITIAL LUNG DISEASE								

Table S2. Taxonomy of the OTUs identified in the study.							
Kingdom	*Phylum*	*Class*	*order*	*family*	*genus*	*species*	Nº OTUS
Fungi							1
	Basidiomycota	*Agaricomycetes*	*Agaricales*				1
			Polyporales	*Fomitopsidaceae*	*Auriporia*	*A. aurulenta*	1
		Agaricostilbomycetes	*Agaricostilbales*	*Agaricostilbaceae*	*Sterigmatomyces*	*S. elviae*	1
		Cystobasidiomycetes	*Cystobasidiales*	*Cystobasidiaceae*	*Cystobasidium*	*C. pinicola*	1
			ord Incertae sedis	*Buckleyzymaceae*	*Buckleyzyma*	*B. aurantiaca*	1
		Microbotryomycetes	*Sporidiobolales*				1
				Sporidiobolaceae	*Rhodosporidiobolus*	*R. colostri*	1
				Incertae_sedis	*Rhodotorula*	*R. mucilaginosa*	6
						R. diobovata	1
						R. nothofagi	1
		Tremellomycetes	*Filobasidiales*	*Foilobasidiaceae*	*Filobasidium*	*F. magnum*	1
			Tremellales	*Tremellaceae*			1
					Naganishia	*N. albida*	5
					Tremella	*T. indecorata*	1
						T. phaeophysciae	1
					Cryptococcus	*Cr. uniguttulatus*	1
						Cr. neoformans	1
				fam Incertae sedis	*Kwoniella*	*K. dendrophila*	1
			Cystofilobasidiales	*Cystofilobasidiaceae*	*Cystofilobasidium*	*C. capitatum*	1
				fam Incertae sedis	*Kwoniella*	*K. dendrophila*	1
		Ustilaginomycotina_cls	*Malasseziales*	*Malasseziaceae*	*Malassezia*	*M. globosa*	2
						M. restricta	1
						M. sympodialis	1
		Wallemiomycetes	*Wallemiales*	*Wallemiaceae*	*Wallemia*	*W. sebi*	2
	Ascomycota						3
		Dothideomycetes	*Capnodiales*	*Cladosporiaceae*	*Cladosporium*		10
			Dothideales				1
				Dothioraceae	*Aureobasidium*	*A. pullulans*	1
			Pleosporales				1
		Leotiomycetes	*Erysiphales*	*Erysiphaceae*	*Golovinomyces*	*G. leucheriae*	1
		Saccharomycetes	*Saccharomycetales*				1
				fam Incertae sedis	*Debaryomyces*	*D. mycophilus*	1
					Candida	*C. parapsilosis*	1
						C. zeylanoides	1
					Wickerhamomyces	*W. anomalus*	1
				Metschnikowiaceae	*Clavispora*	*C. lusitaniae*	1
				Saccharomycetaceae	*Torulaspora*	*T. delbrueckii*	1
					Saccharomyces	*S. cerevisiae*	1
		Sordariomycetes	*Hypocreales*	*fam Incertae sedis*			1
				Nectriaceae	*Fusarium*	*F. pseudensiforme*	1
				Hypocreaceae	*Trichoderma*		1
		Eurotiomycetes	*Chaetothyriales*				2
			Eurotiales	*Trichocomaceae*	*Aspergillus*	*A. flavus*	1
						A. subversicolor	1
					Penicillium	*P. polonicum*	1
	Chytridiomycot	*Rhizophlyctiomycetes*	*Rhizophlyctidales*	*Rhizophlyctidaceae*	*Rhizophlyctis*	*R. rosea*	1
		Spizellomycetes	*Spizellomycetales*	*Spizellomycetaceae*	*Powellomyces*		1

Table S3. Cultures: Number of isolates of each fungal species obtained from the culture of MA (green columns) and BAL samples (white columns) in PDA and SDA.

Fungal species	1	2	3	3	4	5	6	6	7	9	10	11	12	16	16	17	18	18	19	20	22	23	23	24	26	27	30	32	33	34	34	37	38	39	40	41	41	42	42	43	43	44	47	49	50	51	TOTAL
Alternaria sp				1				2																	2	1											3		7		2				4		22
Alternaria alternata/tenuisima																															1						4		4								9
Alternaria arborescens																										5											1		2								8
Alternaria chlamydosporia																															16																16
Aspergillus sp							1	101				1	1							1	1					4	2										20		4						3		139
Aspergillus niger													1												6	3	2																				12
Aureobasidium sp																										48																					48
Aureobasidium pullulans				4				1			6		1					**22**							**1**	**144**	**10**	**67**			**99**						**9**		**1**		**2**				6		373
Candida sp			2	136					9		6							**12**																													165
Candida parapsilosis											1		8										**300**	**20**																					200		529
Cladosporium sp				1																			**52**	**46**	**12**	**117**		**3**			**106**														64		401
Cryptococcus sp				991					220	100																																					1311
Cryptococcus albidus								1				2																																	1		4
Cryptococcus laurentii				80									1																																		81
Cryptococcus neoformans s.l.				25													1	**4**	2																												32
Cystobasidium sp			1																					**1**		**2**	2	2			3																11
Filobasidium sp																																													1		1
Filobasidium magnum																		100								25																					125
Filobasidium uniguttulatum				13																																											13
Fusarium sp																		17							2		**1**				10																30
Fusarium oxysporum																							9																								9
Naganishia sp			2		1																															1											4
Naganishia diffluens												2	1										8		7	526	5	1	24		5						10		2	4							595
Penicillium sp				4									6		**147**				1		1	1	**56**	**1**			17	1			**2**								30		**3**				3		273
Penicillium glabrum																								1	12												3		17							1	34
Rhodotorula sp	1	1		89									2		**1**			**34**					**8**	**1**	**118**	**3**	**9**	**54**	1		**80**	6					**1**		**117**		**123**				4		653
Rhodotorula mucilaginosa				20		3				200													**30**		**25**	**13**																			2		293
Rhodotorula dairenensis																												181									25										206
Rhodotorula diobovata																								**1**			**2**	**59**																			62
Trichoderma sp															5										1																						6
Trichosporon sp				2				1					1										2			2		2			2						3		2		1				1		19
Zigomycetes																										2																					2
Fungi not identified	2				1	1				158	10	30	55		82			261					191	6	471	187	3469	789	1		77						327	19	336	50	85		40	40			6688

PROJECT WORK FLOW

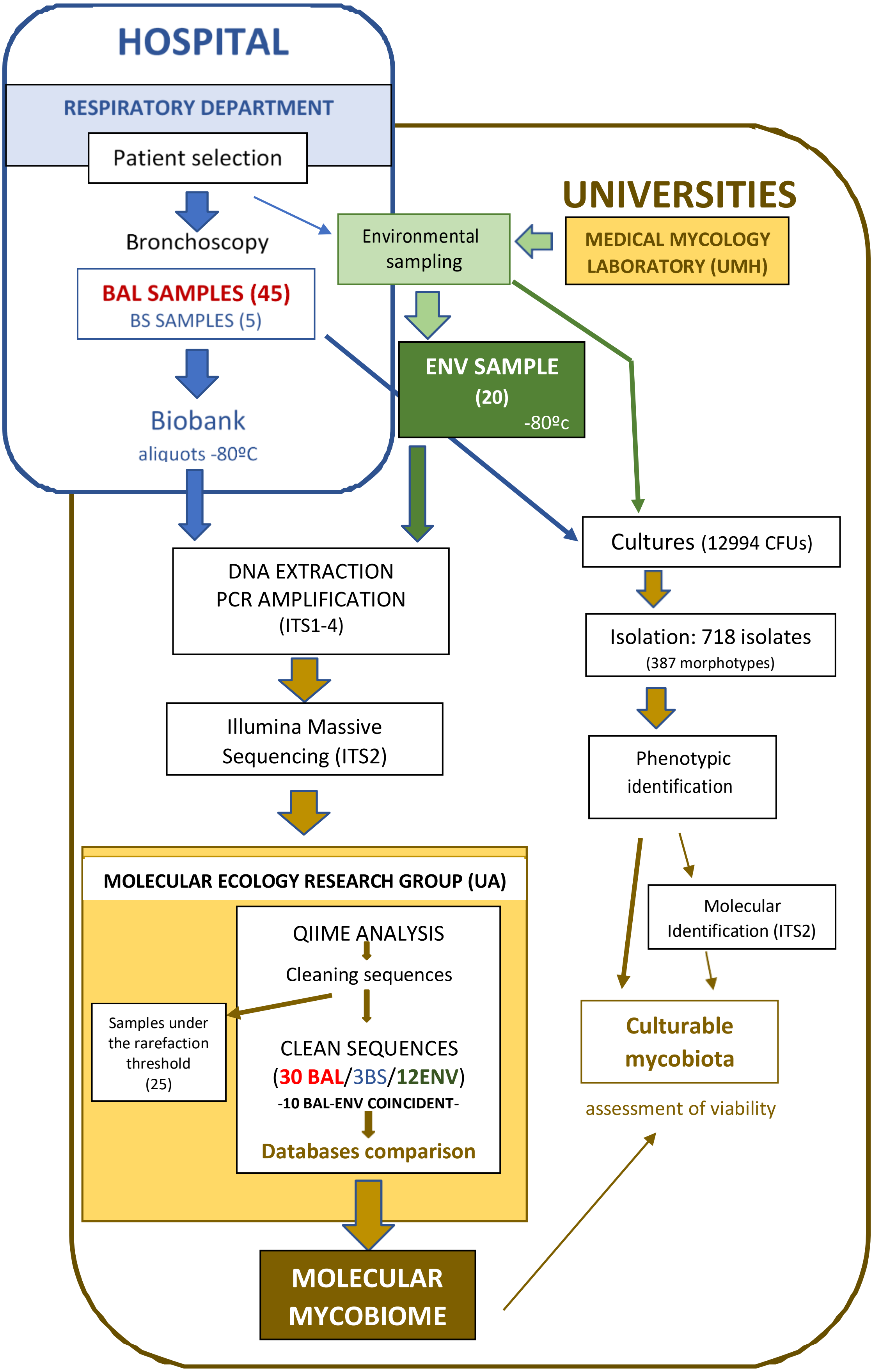

Figure S1: Project workflow

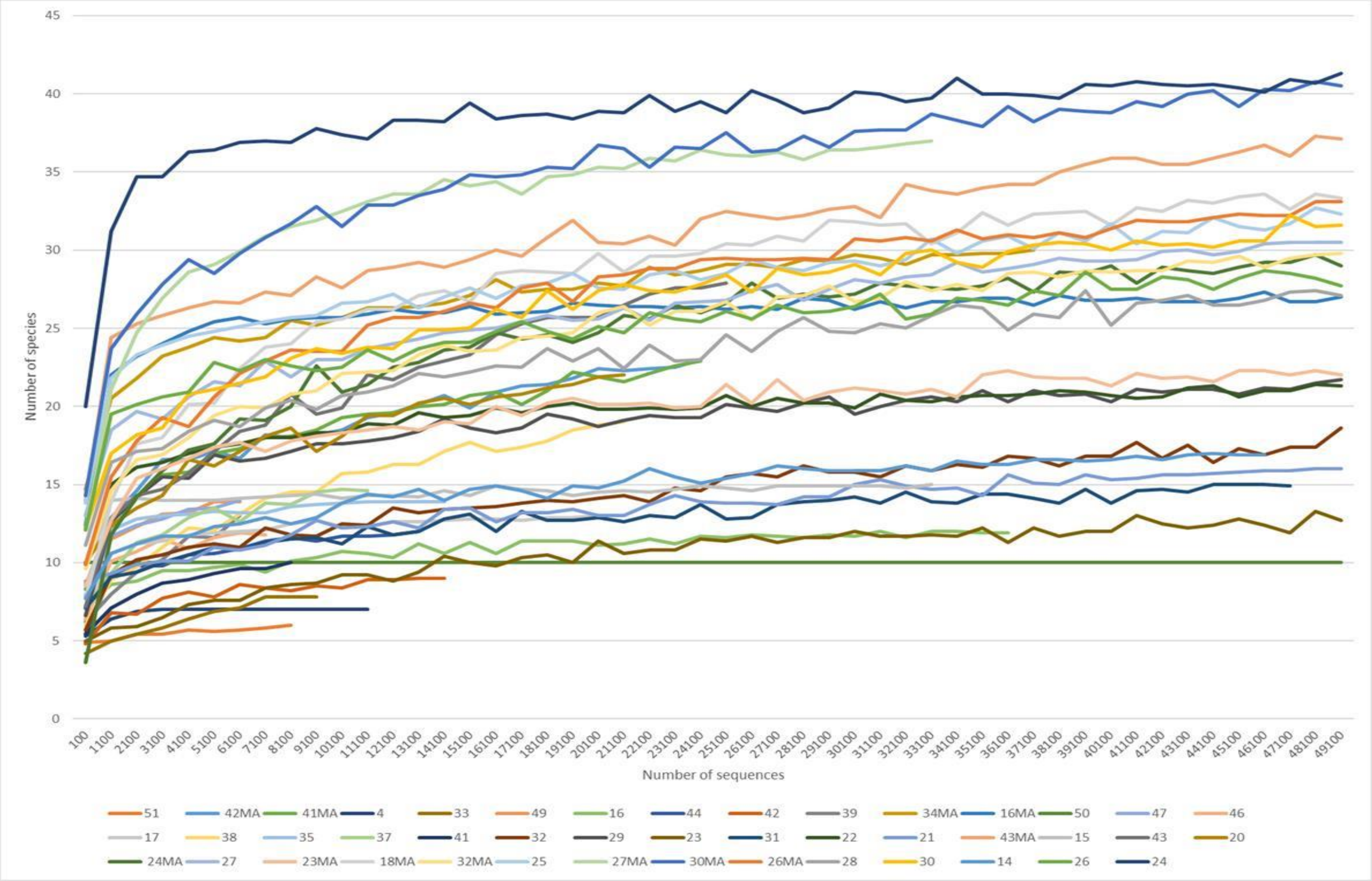

Figure S2: Rarefaction curves

Head of research in each hospital:
ELDA HOSPITAL: Dr. David Orts; ALICANTE HOSPITAL: Dra. Cleofé Fernández; VINALOPÓ HOSPITAL: Dra. Beatriz Amat; VALENCIA CLINICO HOSPITAL: Dr. José Franco

RESPIRATORY TRACT MYCOBIOME: RELATIONSHIP WITH THE ENVIRONMENT

PATIENT DATA SHEET

INSTRUCCIONS

Once the informed consent has been signed, the patient will be assigned a number that will be the result of adding to the hospital's postal code, three digits consecutively and in ascending order, starting with 01. Thus, for example, the first patient at Elda hospital will be patient number: 03600-001
In the bronchoscopy unit of each respiratory department involved, a list will be kept with the identification of each patient in case it is necessary, but only the patient's number will be shown on the data collection sheet as a unique identifier.

The study is part of the Research Project funded by **RESPIRA. Fundación Española del Pulmón (SEPAR) (Spanish Association of Pneumologists)**

The following entities and researchers are collaborating in the study

1. **Universidad Miguel Hernández:**
   Laboratorio de Micología Médica: MF. Colom y C. Ferrer
2. **Universidad de Alicante:**
   Laboratorio de Microbiología: J. Antón y E. Rubio
3. **Hospital Universitario del Vinalopó**
   Servicio de neumología: B. Amat; B. Gálvez
4. **Hospital General Universitario de Alicante**
   Servicio de neumología: C. Fernández
5. **Hospital General Universitario de Elda:**
   Servicio de Neumología: D. Orts y E. Llorca
6. **Hospital Universitario Clínico de Valencia:**
   Servicio de Neumología: J. Franco y V. Esteban

Head of research in each hospital:
ELDA HOSPITAL: Dr. David Orts; ALICANTE HOSPITAL: Dra. Cleofé Fernández; VINALOPÓ HOSPITAL: Dra. Beatriz Amat; VALENCIA CLINICO HOSPITAL: Dr. José Franco

PULMONARY FUNGAL MICROBIOTA STUDY

DATA COLLECTION SHEET

PATIENT NUMBER SEX: M F

					-			

AGE (years)

DATE OF THE TEST (dd/mm/yyyy)

		-			-				

PLACE OF BIRTH (town/province/country)

PLACE OF RESIDENCE (town/province/country): SINCE YEAR:

PROFESSION/SPECIAL RISK ACTIVITIES (exposure to birds, plant detritus, industrial contaminants):

HAVE YOU TRAVELLED OUTSIDE SPAIN? ☐ YES ☐ NO

- PLACE: YEAR:
- PLACE: YEAR:
- PLACE: YEAR:

PNEUMOLOGICAL DIAGNOSIS (check all that apply) :

	COPD		PULMONARY METASTASES
	BRONCHIECTASIAS		ENDOBRONCHIAL FOREIGN BODY
	ILD		
	BRONCHOGENIC CARCINOMA		
	OTHERS (specify):		

Head of research in each hospital:
ELDA HOSPITAL: Dr. David Orts; ALICANTE HOSPITAL: Dra. Cleofé Fernández; VINALOPÓ HOSPITAL: Dra. Beatriz Amat; VALENCIA CLINICO HOSPITAL: Dr. José Franco

IMMUNEDEPRESSION: YES NO
DIAGNOSTIC CODE ID (see below):

IF OTHERS PLEASE, SPECIFY:

ANTIMICROBIAL DRUGS CONSUMED IN THE LAST MONTH (DOSE AND TIME):

BRONCHUS IN WHICH THE BAL SAMPLE IS TAKEN:

AMOUNT INSTILLED:
QUANTITY COLLECTED:
QUANTITY PROCESSED FOR STUDY:

IMMUNO-DEPRESSION CODES	
01	Neutropenia (< 500/mm^3) lasting > 10 days in the last moth
02	Prolonged steroid treatment (> 20 mg/day for > 3 weeks or > 700 mg prednisone in total in the last 2 months
03	Immunosuppressive treatment during the last 3 months
04	Solid organ transplant carrier
05	Hematopoietic transplant carrier.
06	Hematological neoplasia in the last year
07	Non-hematological neoplasia in the last year
08	Chemotherapy treatment in the last 6 months
09	OTHER (please, specify)
